# Poverty prediction using E-commerce dataset and filter-based feature selection approach

**DOI:** 10.1038/s41598-024-52752-7

**Published:** 2024-02-07

**Authors:** Dedy Rahman Wijaya, Raden Ilham Fadhilah Ibadurrohman, Elis Hernawati, Wawa Wikusna

**Affiliations:** School of Applied Science, Telkom University, Bandung, Indonesia

**Keywords:** Computer science, Scientific data

## Abstract

Poverty is a problem that occurs in many countries, notably in Indonesia. The common methods used to obtain poverty information are surveys and censuses. However, this process takes a long time and uses a lot of human resources. On the other hand, governments and policymakers need a faster approach to know social-economic conditions for area development plans. Hence, in this paper, we develop e-commerce data and machine learning algorithms as a proxy for poverty levels that can provide faster information than surveys or censuses. The e-commerce dataset is used and this high-dimensional data becomes a challenge. Hence, feature selection algorithms are employed to determine the best features before building a machine learning model. Furthermore, three machine learning algorithms such as support vector regression, linear regression, and k-nearest neighbor are compared to predict the poverty rate. Hence, the contribution of this paper is to propose the combination of statistical-based feature selection and machine learning algorithms to predict the poverty rate based on e-commerce data. According to the experimental results, the combination of f-score feature selection and support vector regression surpasses other methods. It shows that e-commerce data and machine learning algorithms can be potentially used as a proxy for predicting poverty.

## Introduction

In the last decades, poverty has been a common problem in developing countries. In March 2018, the Central Bureau of Statistics reported that the number of poor people in Indonesia reached 25.95 million people (9.82 percent). This number decreased by around 633.2 thousand people compared to September 2017 of 26.58 million people (10.12 percent)^1^. The data was obtained by the Central Bureau of Statistics by conducting a national socio-economic survey commonly abbreviated as SUSENAS. It is a household-based survey that collects information on socio-economic characteristics such as education, health, family planning, travel information, crime, housing, social protection, and household consumption and expenditure^2^. SUSENAS survey can be estimated to take a long time and there will be a period between one survey and another. In terms of cost, it is also very expensive. Hence, how the government estimates poverty to achieve better program targets is not an easy task. On the other hand, the digital revolution continues to generate abundant data, which provides new opportunities to capture information about socio-economic conditions at various levels of abstraction to summarize development progress. These data can be used to monitor changes in the prosperity level, as well as to measure the impact of government programs. One prospective data source to capture socio-economic conditions is e-commerce data. The e-commerce market in Indonesia is one of the largest in Southeast Asia with a contribution of up to fifty percent of all transactions in the Southeast Asian region. The growth of the population of internet users can increase e-commerce penetration in Indonesia so that its contribution to the economy has the potential to continue to increase. Even without taking into account the B2B service sector, the gross merchandise value of the e-commerce market in Indonesia is projected to grow around eight times by 2022^3^.

In the last few years, several data sources have been reported for poverty estimation such as satellite imagery and call detail records (CDRs)^4–7^. However, these datasets have assumptions for example the light intensity of nightlight data from satellite imagery reflects high economic activity in a particular area. Moreover, high mobile phone credit is related to welfare in CDRs data. In contrast, e-commerce data can reflect the real expenditure for necessities at the household level without assumptions^8^. Thus, this dataset has more complied with the formal calculation of poverty level. Nevertheless, the study of e-commerce data for poverty prediction is relatively new and rare. Furthermore, the one challenge of e-commerce data utilization is high-dimensional data that must be reduced for performance improvement of machine learning algorithms. Our hypothesis is a feature subset produced by feature selection algorithm can improve the performance of machine learning algorithm.

According to this background, we propose a solution to complement survey and census by using e-commerce data and machine learning algorithms, especially in Indonesia. The proposed method can be used as a fast and low-cost solution to predict the poverty level. It can be used by governments and policymakers as a baseline to determine development policies. The e-commerce dataset contains the calculation of the number of purchases from a particular area, so, it can be seen in that region whether the area is prosperous or vice versa. Thus, the contribution of this research is to propose the combination of statistical-based feature selection and machine learning algorithms to build a model for predicting poverty in Indonesia using a dataset that represents Indonesian people’s needs based on e-commerce data. In this study, we have several motivations as follows:

1. This study used e-commerce data from one of the largest e-commerce companies in Indonesia. By using the e-commerce data, the data source can be rapidly updated to complement the National Socio-economic Survey that records poverty every 5 years. Real-time poverty prediction can help governments and policymakers to determine the priority of development plans.
2. Existing studies utilized several methods to predict poverty such as using phone records^9^, satellite imagery^10,11^, and small area estimation^12^. However, these studies used several assumptions to predict poverty. On the other hand, we use an e-commerce dataset obtained from one of the e-commerce platforms in Indonesia. We argue that e-commerce data can represent the economic conditions in a particular area.

Hence, to the best of our knowledge, the study of poverty estimation using e-commerce data and machine learning algorithms is relatively new and rare. In our previous works, we have performed poverty prediction using machine learning algorithms. However, it only uses one feature selection algorithm that makes the study quite limited^8,13^. In addition, wrapper-based feature selection algorithm were also utilized, but it could not provide satisfactory results^14^. Hence, to emphasize the originality, in this paper, we use three statistical-based feature selection and three machine learning algorithms to find the best model for poverty estimation. This approach not only can be used for Indonesian data but also potentially adopted for e-commerce data from other countries.

## Methods

The dataset used in this research is sample advertising data from one of the e-commerce companies in Indonesia which was regenerated and changed in value. There are eight items such as motorbikes, cars, apartments for sale, apartments for rent, houses for sale, houses for rent, land for sale, and land for rent in 2016. To measure the level of poverty, a poverty limit/line is needed. The poverty line reflects the rupiah value of the minimum expenditure a person needs to fulfill his basic life needs a month, both food and non-food needs. These items are included in the list of basic needs commodities^15^. The advertisements used for this study are from Java Island which is the most contributed island for posting the advertisement in total 18,881,913 advertisements in 118 cities/districts. Table 1 shows the detail of the dataset used in this study.

**Table 1:** Dataset description.

Items	Number of advertisements
Cars	6,933,513
Motorbikes	6,313,016
House for sale	3,594,545
House for rent	336,758
Apartment for sale	250,504
Apartment for rent	259,689
Land for sale	1,179,972
Land for rent	13,916

**Figure 1:**
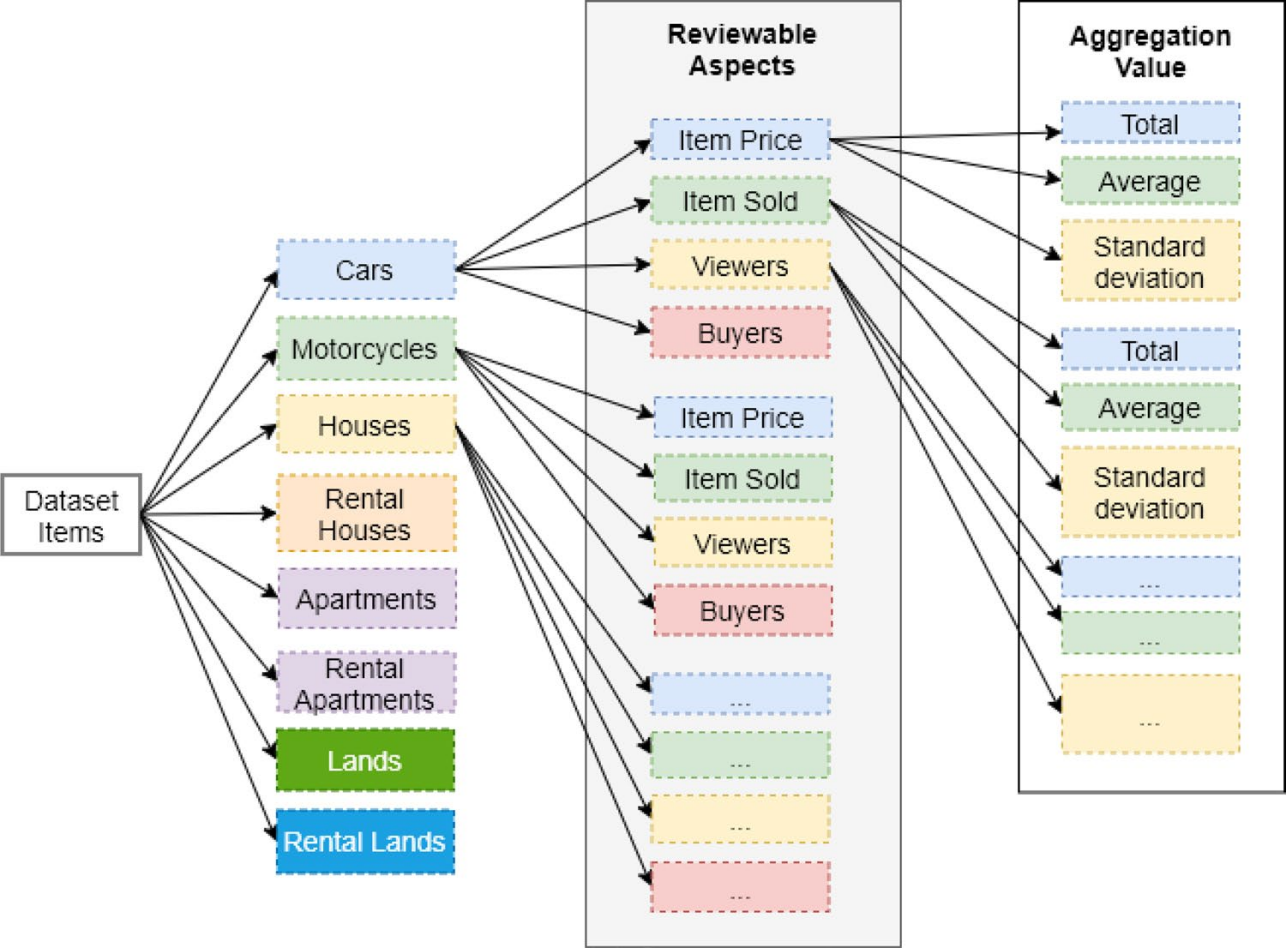
Illustration of items and aspects extraction from dataset.

From each item in Table 1, four aspects were aggregated per city such as the number of items sold, selling price, number of buyers, and number of viewers for three statistical features (average, sum, and standard deviation). Initially, the used e-commerce dataset contains 96 numeric features and an actual poverty rate as a continuous label. The ground truth of poverty rate was referred to the official poverty rate issued by BPS (Statistics Indonesia) in the current year. Thus, we have 8 items × 4 aspects × 3 statistical features = 96 features according to this way as shown in Fig. 3. Our dataset contains 96 columns (features) × 118 rows (cities). The extraction of items and aspects from the dataset is shown in Fig. 1. Because the data dimension is relatively huge, we used statistical-based feature selection algorithms to select the most relevant features and machine learning algorithms to train and build prediction models using data from the selected feature. Generally, the prediction process using machine learning algorithms and statistical-based feature selection has five stages. It is started with pre-processing data, normalization, feature selection, training model, and evaluation.

**Figure 2:**
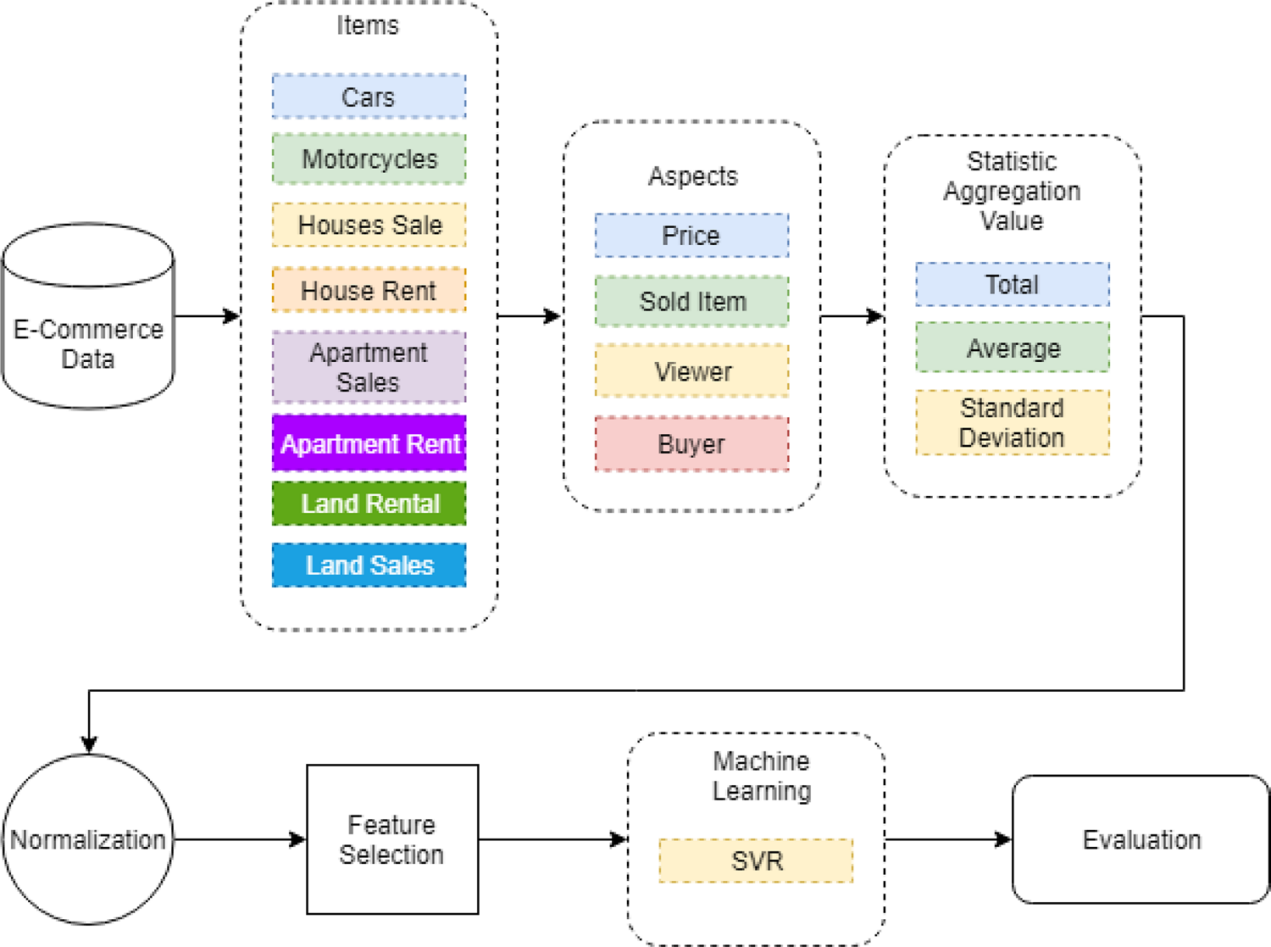
Flow of the proposed method.

Figure 2 shows the flow of the proposed method. It starts by extracting e-commerce data into items, aspects, and calculates the statistic aggregation values. The extracted data still contains dirty data. Thus, the dirty data needs to be cleaned up. Clean data will be normalized to be scaled to the same scale. Data normalization is necessary to uniform the scale. So, it is converted into [0, 1]. In this research work, the min–max normalization method is used for the normalization method. The min–max method is one of the normalization techniques to standardize the dataset using linear transformation^16^. This normalization method transforms e-commerce data into a fixed range. This normalization method ensures that a huge data range is constrained within a specific range. It transforms a value *X*_*0*_ to X_p_ which fits in the specified range. The criteria are given by Eq. (1),

1 $${X}_{p}=\frac{{X}_{o}-{\text{min}}(x)}{{\text{max}}\left(x\right)-{\text{min}}(x)}$$

where *X*_*p*_ is the new value for variable *X, X*_*O*_ is the current value for variable *X. min(x)* and *max(x)* are the minimum and the maximum data points in the dataset, respectively. We used min–max normalization because of its performance for having less number of misclassification errors. Also, it has been reported for satisfactory performance in supervised and unsupervised learning^17–19^.

### Feature selection algorithms

Many datasets have a high dimension such as the marketplace, healthcare, social media, etc. However, these high-dimensional data cause a problem for the algorithm that was designed for low-dimensional space. They can also increase the memory usage of the computer. To deal with high-dimensional data, this paper uses several filter-based feature selection algorithms such as f-score, chi-square, and correlation-based feature selection (CFS). The filter method does not rely on any learning algorithm. They rely on data characteristics to assess the importance of features. Filter methods are usually more computationally efficient than other methods^20^.

F-score is known as a simple technique for measuring discrimination of two sets of real numbers^21^. Given training vector *x*_*k*_*, k* = 1, 2,…, m, if the number of positive and negative instances are n_+_ and n_–_ respectively, then f-score of the ith feature is defined as Eq. (2),

2 $${F}_{i}= \frac{{\left({\overline{x} }_{i}^{\left(+\right)}- {\overline{x} }_{i}\right)}^{2}+ {\left({\overline{x} }_{i}^{\left(-\right)}- {\overline{x} }_{i}\right)}^{2}}{\frac{1}{{n}_{+}-1{\sum }_{k=1}^{n+}{\left({x}_{k,i}^{\left(+\right)}-{\overline{x} }_{i}^{+}\right)}^{2}}+ \frac{1}{{n}_{-}-1}{\sum }_{k,i}^{n-}{\left({x}_{k,i}^{\left(-\right)}-{\overline{x} }_{i}^{-}\right)}^{2}}$$

where ${\overline{x} }_{i}^{\left(+\right)}$, ${\overline{x} }_{i}$, $\overline{x }$ are the average of the *i*th feature of the whole, positive, and negative datasets, respectively. The numerator indicates the discrimination between the positive and negative sets, and the denominator indicates the one within each of the 2 sets. The greater f-score value indicates the feature is more discriminative.

Chi-square utilizes the test of independence to assess if the feature is independent of the class label^22^. Chi-square criteria can be defined in Eq. (3),

3 $${x}^{2}= \sum_{j=1}^{2}\sum_{s=1}^{c}\frac{{({n}_{js}- {\mu }_{js})}^{2}}{{\mu }_{js}}$$

where c = number of classes, ${n}_{js}$ = number of patterns in the *j*th interval, *s*th class, ${R}_{i}$= number of patterns in the *j*th interval = ${\sum }_{s=1}^{c}{n}_{js}$, ${K}_{s}$= number of patterns in the *s*th class = ${\sum }_{j=1}^{2}{n}_{js}$, $N$ = total number of patterns = ${\sum }_{j=1}^{2}{R}_{i}$, ${\mu }_{js}$ = expected frequency of ${n}_{js}$ = ${R}_{i} \times \frac{{K}_{s}}{N}.$

If ${R}_{i}$ and ${K}_{s}$ is 0, ${\mu }_{js}$ is set to 0.1. A higher chi-square value indicates that the feature is relatively important. However, Chi-Square algorithm needs discrete values to perform feature selection. Hence, in this experiment, the poverty rate as continuous values is rounded to get discrete values. Moreover, the basic idea of CFS algorithm is to use a correlation-based heuristic to evaluate the worth of a feature subset^23^. The CFS criteria are defined in Eq. (4),

4 $${merit}_{s}= \frac{k\overline{{r }_{cf}}}{\sqrt{k+k(k-1)}\overline{rff} }$$

where the score shows the heuristic “merit” of a feature subset* s* containing *k* features, $\overline{{r }_{cf}}$ is the mean of class correlation where $f \in s$, $\overline{rff }$ is the average feature intercorrelation. The basic idea is the stronger correlation with the class label and the weaker intercorrelated to each other is the better feature subset.

### Machine learning algorithms

Mainly, in this research, we used a support vector regression (SVR) algorithm because the SVM for classification already showed good results in medical diagnostics, optical character recognition, electric load forecasting, and other fields^24^. Also, the SVR algorithm is the most common application form of SVMs to build a machine learning regression model^25,26^. The advantages of SVM are a unique solution, not sensitive to small changes of parameters, and providing increased performance^27^. In addition, the SVM is a machine learning algorithm that implements structural risk minimalization to obtain good generalization on a limited number of learning patterns^28^. The data from several stages before will be used to train in this stage. Considering a training dataset, {($\overrightarrow{{x}_{1}},{z}_{1}$), …, ($\overrightarrow{{x}_{i}},{z}_{i}$)} that corresponds to features where $\overrightarrow{{x}_{i}}, {z}_{i}$ are feature vector and target output, respectively. The standard criteria of SVR are given in Eq. (5) through Eq. (9)^29^.

5 $$\underset{w,b,\xi ,{\xi }^{*}}{{\text{min}}}\mathit{ }\frac{1}{2}{w}^{t}w+C\sum_{i=1}^{l}{\xi }_{i}+ C\sum_{i=1}^{l}{\xi }_{i}^{*}$$

6 $$\begin{aligned} {\text{subject to}}\,\,\, & w^{t} \emptyset \left( { x_{i} } \right) + b - z_{i} \le \varepsilon + \xi_{i} , \\ & z_{i} - w^{t} \emptyset \left( {x_{i} } \right) - b \le \varepsilon + \xi_{i}^{*} , \\ & \xi_{i} , \xi_{i}^{*} \ge 0,\quad i = 1, \ldots ,l. \\ \end{aligned}$$

where $w, C, \xi ,\varepsilon ,$ and $b$ as slope matrix, regularization parameter slack variable for soft margin, tolerance margin, and the intercept/bias, respectively. The dual problem is

7 $$\underset{\propto ,{\propto }^{*}}{\mathrm{min }}\frac{1}{2}{\left(\alpha - {\alpha }^{*}\right)}^{t}Q\left(\alpha - {\alpha }^{*}\right)+\varepsilon \sum_{i=1}^{l}\left({\alpha }_{i} - {\alpha }_{i}^{*}\right) +\sum_{i=1}^{l}{z}_{i}\left({\alpha }_{i} - {\alpha }_{i}^{*}\right)$$

8 $$\begin{aligned} {\text{subject to}}\,\,\,\, & e^{t} \left( {\alpha {-} \alpha^{*} } \right) = 0, \\ & 0 \le \alpha_{i} ,\alpha_{i}^{*} \le C,\quad i = 1, \ldots , l, \\ \end{aligned}$$

where $\alpha - {\alpha }^{*}$ denotes Lagrangian multipliers. ${Q}_{ij}=K\left({x}_{i},{x}_{j}\right)\equiv \varnothing {\left({x}_{i}\right)}^{t}\varnothing \left({x}_{j}\right).$ The approximate function after solving the problem in Eq. (8) is

9 $$\sum_{i=1}^{l}\left({-\alpha }_{i}+ {\alpha }_{i}^{*}\right)K\left({x}_{i}, x\right)+b$$

the output from the model is ${\alpha }^{*}- \alpha$.

To ensure the model made has good parameter values, in this experiment, grid search was performed to determine kernel between RBF and polynomial, epsilon value within [0.1, 0.5, 1.0, 1.5, 2.0], parameter C within [1, 10, 100, 1000], and gamma within [0.001, 0.0001]. LIBSVM was used as the library for SVR. From grid search, we determined to use kernel, epsilon, C, and gamma parameters are RBF, 0.5, 10, 0.001, respectively.

Also, in this research, we used k-nearest neighbor regression (k-NN) and linear regression (LR) to compare with SVR performance. The k-NN algorithm is a method for classifying objects based on the closest training example in feature space^30^. K-NN is a type of lazy learning where the function is only approximated locally. The same method can be used for regression by assigning the property value for the object to be the average of the values of its K nearest neighbor. K-NN is widely adopted for classification and regression because of its simplicity and intuitiveness^31^. While LR is used to study the linear relationship between a dependent variable and one or more independent variables^32^.

### Evaluation

For evaluation, the leave-one-out method was used for cross-validation. In this experiment, we used the root mean squared error (RMSE) and R-squared (R^2^) to measure the performance of the machine learning model. To measure the error between actual data and prediction vectors, the RMSE is used. The best prediction results are obtained if the RMSE value is low. It means the difference between actual and prediction data is low. Equation (10) shows the equation of RMSE,

10 $$RMSE\left(y, \widehat{y}\right)= \sqrt{\frac{{\sum }_{i=1}^{L}{({y}_{i}- {\widehat{y}}_{i})}^{2}}{L}}$$

where, $y, \widehat{y}, L$ indicate actual value, prediction value, and data length, respectively. In addition, to measure the performance, we used R^2^ as shown in Eq. (11) to show the parts of the variance of the actual data. R^2^ will assess the regression model and whether the model can correctly predict the actual value. The R^2^ value will be ranged from 0 to 1 and if the value is nearly 1 or even 1, it means the model is almost perfect in predicting the actual data. Otherwise, if the value equals 0 or negative, it means the model does not follow the trend of the actual data^33^.

11 $${R}^{2}\left(y, \widehat{y}\right)= 1- \frac{\sum_{i=1}^{L}{({y}_{i}- {\widehat{y}}_{i})}^{2}}{\sum_{i=1}^{L}{({y}_{i}- {\overline{y} }_{i})}^{2}}$$

## Results and discussion

F-score, chi-square, and CFS feature selection are used to select some of the most relevant features. The feature selection was used to rank all the features in the dataset. The result of this stage is to rank the feature indexes. Tables 2 and 3 is feature selection result for f-score and chi-square, respectively. Also, we found that CFS produced an inconsistent index ranking in every experiment. The experimental results are shown in Table 4.

**Table 2:** F-score feature ranking.

Ranks	Ranked feature indexes
1–10	49, 60, 62, 63, 37, 13, 14, 61, 38, 50
11–20	69, 84, 66, 86, 48, 85, 12, 36, 0, 1
21–30	57, 54, 3, 76, 51, 75, 2, 77, 6, 64
31–40	9, 15, 56, 18, 22, 82, 83, 21, 24, 87
41–50	10, 39, 42, 70, 45, 79, 71, 25, 20, 67
51–60	72, 68, 23, 94, 91, 34, 8, 59, 92, 65
61–70	11, 80, 27, 35, 93, 89, 26, 31, 95, 44
71–80	32, 88, 47, 55, 90, 52, 16, 17, 30, 73
81–90	7, 4, 53, 33, 43, 46, 5, 19, 41, 28
91–96	81, 78, 40, 58, 29, 74

**Table 3:** Chi-square feature ranking.

Ranks	Ranked feature indexes
1–10	36, 63, 60, 48, 57, 54, 51, 6, 9, 66
11–20	18, 15, 69, 38, 3, 13, 0, 24, 21, 27
21–30	33, 30, 42, 45, 87, 72, 84, 14, 75, 12
31–40	93, 39, 61, 65, 62, 90, 86, 89, 50, 49
41–50	71, 52, 85, 78, 44, 81, 64, 37, 74, 68
51–60	2, 67, 73, 47, 1, 20, 53, 25, 88, 59
61–70	70, 26, 31, 34, 8, 79, 95, 82, 56, 23
71–80	28, 11, 94, 76, 32, 43, 83, 55, 58, 10
81–90	92, 77, 91, 80, 29, 46, 19, 7, 35, 22
91–96	16, 40, 4, 17, 41, 5

**Table 4:** CFS feature ranking experiment.

Ranks	Ranked feature indexes		
	1st Experiment	2nd Experiment	3rd Experiment
1–10	95, 94, 93, 91, 90, 89, 18, 46, 71, 66	95, 94, 93, 91, 90, 89, 74, 61, 71, 6	95, 94, 93, 91, 90, 89, 61, 3, 16, 62
11–20	42, 63, 64, 87, 62, 72, 32, 10, 50, 22	34, 1, 19, 4, 52, 51, 70, 27, 76, 48	54, 65, 9, 59, 70, 78, 23, 43, 45, 67
21–30	67, 86, 70, 26, 57, 21, 14, 11, 12, 2	15, 63, 49, 12, 75, 84, 55, 81, 29, 28	52, 73, 84, 15, 55, 11, 40, 10, 83, 46
31–40	58, 56, 83, 92, 30, 19, 52, 74, 81, 69	21, 58, 24, 16, 23, 33, 36, 83, 2, 35	56, 38, 63, 75, 0, 21, 74, 66, 77, 34
41–50	6, 44, 40, 24, 23, 82, 75, 0, 29, 7	7, 57, 10, 78, 88, 8, 0, 59, 9, 60	14, 71, 30, 39, 37, 88, 6, 50, 7, 48
51–60	79, 27, 31, 47, 33, 65, 68, 8, 45, 51	65, 5, 67, 85, 56, 11, 77, 42, 47, 38	27, 4, 64, 51, 20, 24, 80, 49, 47, 32
61–70	43, 1, 78, 85, 5, 9, 15, 20, 41, 25	86, 17, 41, 62, 64, 20, 50, 37, 80, 43	1, 76, 57, 86, 81, 85, 17, 53, 68, 42
71–80	73, 4, 80, 34, 61, 60, 17, 88, 55, 35	45, 92, 14, 22, 25, 54, 72, 68, 31, 46	58, 79, 12, 82, 25, 13, 26, 35, 29, 60
81–90	37, 77, 84, 59, 13, 38, 16, 28, 36, 54	53, 44, 82, 30, 26, 66, 3, 13, 40, 18	72, 2, 8, 19, 31, 22, 5, 41, 18, 87
91–96	39, 76, 48, 53, 3, 49	32, 73, 39, 87, 79, 69	28, 92, 33, 36, 44, 69

According to the experiment, the first six feature index is consistent with the ranking, while the others show inconsistency. Thus, we decided that the results of the CFS algorithm cannot be used for building a machine learning model. We only used f-score and chi-square feature selection for building the machine learning model. After the feature is ranked, we also try to find the best result by conducting several prediction experiments from the lowest number of features, starting from 10 features, 20 features, 30 features, 40 features onwards to 96 features. Then, the evaluation process is included in every experiment using R^2^ and RMSE. Also, we used LR and k-NN besides SVR to compare and prove that SVR is the best method. Tables 5 and 6 show SVR prediction results without feature selection and with feature selection, respectively.

**Table 5:** Prediction experiments without feature selection.

ML algorithm	SVR		Linear regression		k-NN	
Column used	R^2^	RMSE	R^2^	RMSE	R^2^	RMSE
10	0.28107	17.438	0.1653	20.246	− 0.49179	36.183
20	0.31722	16.561	− 1.8478	69.074	− 0.51625	36.777
30	0.39354	14.71	− 0.17544	28.511	− 0.10348	26.765
40	0.37034	15.272	− 3.7001	114	− 0.14426	27.754
50	0.3689	15.307	− 32.732	818.18	− 0.10209	26.731
60	0.41782	14.121	− 8.7387	236.21	− 0.19901	29.082
70	0.39643	14.64	− 53.072	1311.5	− 0.13893	27.625
80	**0.42321**	**13.99**	− 103.86	2543.4	− 0.00019416	24.26
90	0.42109	14.042	− 125.98	3079.8	− 0.093847	26.531
96	0.41582	14.169	− 213.48	5202.2	0.013777	23.921
Best Score	**0.42321**	**13.99**	0.1653	20.246	0.013777	23.921

**Table 6:** Prediction experiments with feature selection.

ML algorithm	SVR		Linear regression		k-NN	
Column used	R^2^	RMSE	R^2^	RMSE	R^2^	RMSE
F-score						
10	0.10379	21.738	− 235.07	5725.8	− 0.34956	32.734
20	0.17101	20.107	− 178.36	4350.4	− 0.20913	29.328
30	0.27758	17.522	− 141.12	3447.1	− 0.20955	29.338
40	0.33815	16.053	− 100.4	2459.5	− 0.090936	26.461
50	0.34931	15.783	− 117.58	2876.2	− 0.24026	30.083
60	0.35681	15.601	− 189.72	4626	0.091396	22.038
70	0.33041	16.241	− 130.22	3182.8	− 0.023141	24.816
80	0.31791	16.544	− 206.08	5022.7	− 0.12612	27.314
90	**0.42765**	**13.882**	− 305.58	7436.1	− 0.17697	28.548
96	0.41582	14.169	− 213.48	5202.2	0.013777	23.921
Best score	**0.42765**	**13.882**	− 100.4	2459.5	0.091396	22.038
Chi^2^						
10	0.21749	18.98	− 1.6228	63.615	− 0.40376	34.048
20	0.26046	17.938	− 1.6719	64.806	− 0.30081	31.551
30	0.2988	17.008	− 4.2461	127.24	0.0017002	24.214
40	0.33031	16.244	− 114.01	2789.7	− 0.15016	27.897
50	0.32104	16.468	− 340.86	8291.8	− 0.14628	27.803
60	0.33176	16.208	− 270.71	6590.3	− 0.084942	26.315
70	0.3321	16.2	− 403.41	9809.1	0.08046	22.304
80	0.39998	14.554	− 233.54	5688.7	0.048056	23.089
90	0.4017	14.512	− 162.72	3971.1	− 0.066467	25.867
96	0.41582	14.169	− 213.48	5202.2	0.013777	23.921
Best score	0.41582	14.169	− 1.6228	63.615	0.08046	22.304

Tables 5 and 6 show the results of our experiments in this study. The experiment results are based on the number of features, the machine learning algorithm, and the feature selection algorithm. Bold text indicates the best results. Table 5 shows the comparison of machine learning algorithms without using feature selection and the best R^2^ score obtained by SVR is 0.42321. Table 6 shows the SVR experiments using feature selection and the best R^2^ value is 0.42765 using f-score feature selection and 90 features as shown in Table 2. According to these experimental results, a feature selection algorithm can improve the performance of machine learning to handle high-dimensional data of the e-commerce dataset.

**Figure 3:**
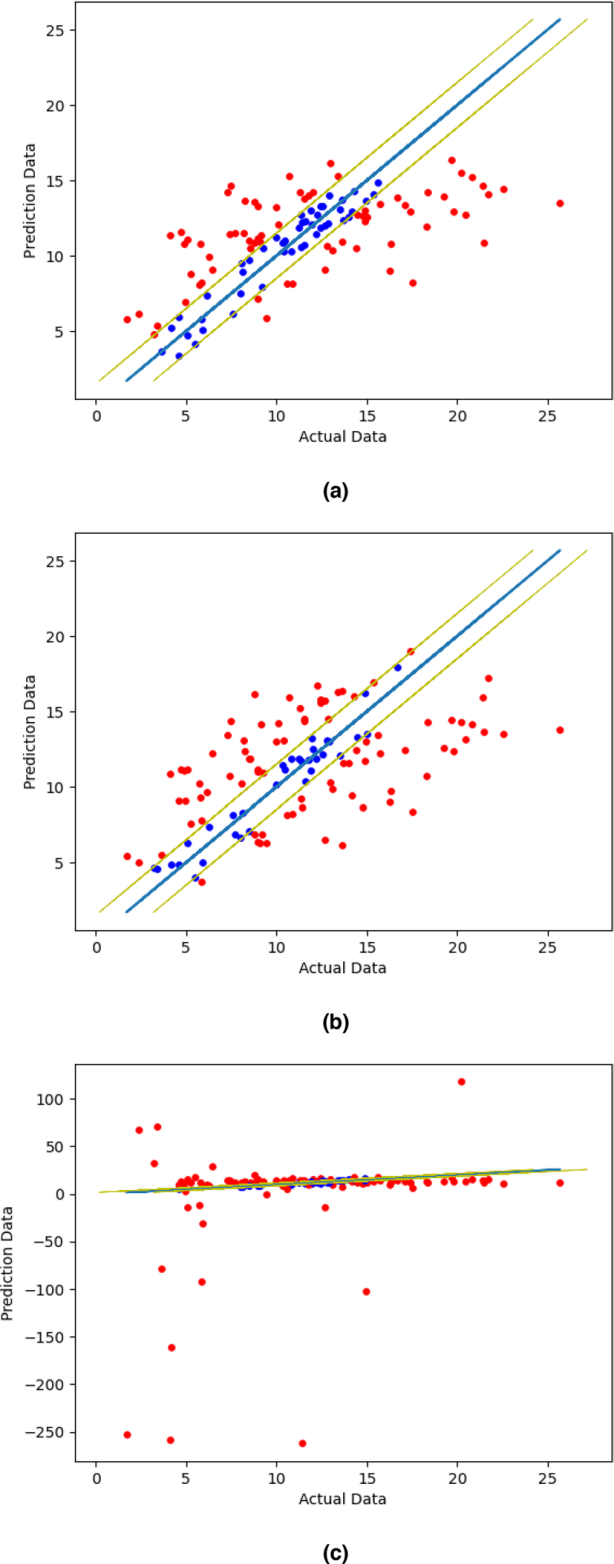
Data visualization of (**a**) SVR, (**b**) k-NN regression, (**c**) linear regression.

The best models of SVR, k-NN regressor, and LR are visualized in Fig. 3a–c, respectively. The blue line and the yellow lines are the trend of actual data (*y* = *x*) and the threshold (± 1.5), respectively. The data between the error margins means the data has less prediction error and vice versa.

The visualized data of SVR result shows more data with less error than other models. Thus, it makes SVR better than other models that we compared in this paper. The worst data visualization is displayed while predicting using linear regression, some data are far from the actual data. It means that LR produces the worst model.

**Figure 4:**
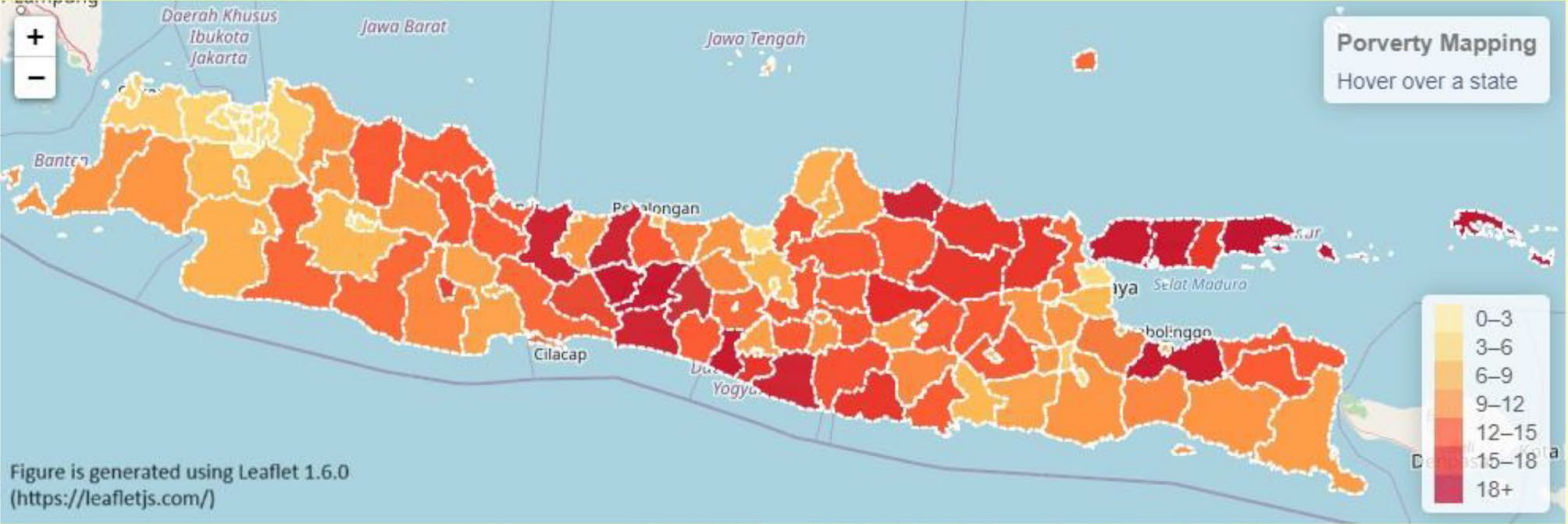
Actual poverty rate mapping in each city in Java island.

**Figure 5:**
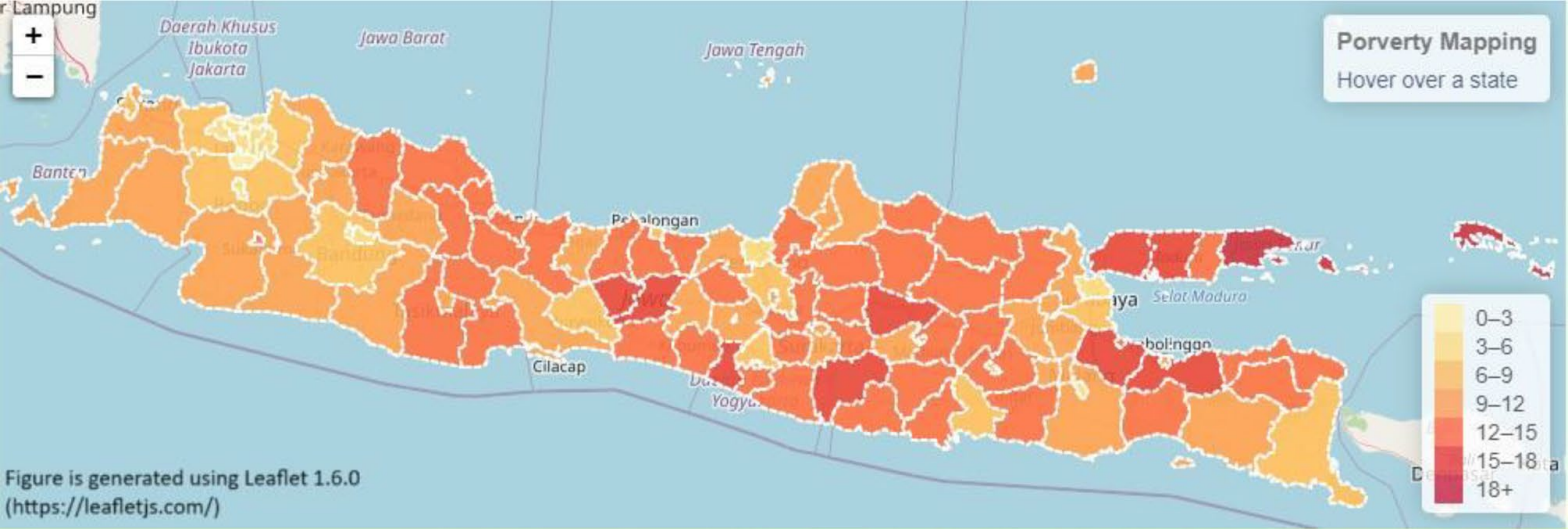
Predicted poverty mapping based on cities on Java island.

Furthermore, the results are also visualized in the choropleth map in Figs. 4 and 5. Figure 4 shows the Java Island actual poverty rate mapping. They were generated using Leaflet 1.6.0^34^. Leaflet is an open source JavaScript library used to build web mapping applications. The darker color indicates a higher poverty rate and vice versa. The predicted poverty mapping is displayed in Fig. 5. From the actual data in Fig. 4, predicted data in Fig. 5 shows a lower level of poverty rate. This result indicates that the prediction model produces underestimation results. Finally, Table 7 presents a detailed comparison between the actual and predicted poverty rates at the city level. The table provides a breakdown of each poverty percentage value for a comprehensive analysis.

**Table 7:** Comparison of actual and predicted poverty rates.

City/district	Actual	Predicted
Kepulauan Seribu	11.4	9.44
Lebak	9.97	11.53
Pacitan	16.68	12.42
Banjar City	7.41	11.33
Sumenep	20.2	11.68
Situbondo	13.63	12.34
Sampang	25.69	12.33
Indramayu	14.98	12.36
Pandeglang	10.43	11.28
Trenggalek	13.39	12.63
Pamekasan	17.41	13.10
Sukabumi	8.96	11.60
Probolinggo City	8.17	11.78
Rembang	19.28	13.18
Probolinggo	20.82	12.91
Brebes	19.79	11.98
Lumajang	11.52	12.76
Tasikmalaya	11.99	13.42
Serang	5.09	11.36
Sumedang	11.36	11.04
Pasuruan City	7.47	12.94
Ciamis	8.98	12.69
Batang	11.27	13.34
Batu City	4.71	11.67
Pemalang	18.3	11.68
Wonosobo	21.45	11.70
Majalengka	14.19	12.12
Subang	12.27	12.12
Tegal City	8.26	12.07
Bangkalan	22.57	12.89
Bondowoso	14.96	11.19
Blora	13.52	13.36
Pekalongan	12.84	12.58
Blitar City	7.29	12.56
Kuningan	13.97	12.12
Banjarnegara	18.37	12.83
Tegal	10.09	12.69
Purworejo	14.27	12.41
Purbalingga	19.7	13.29
Tuban	17.08	12.11
Cianjur	12.21	12.02
Ngawi	15.61	13.26
Cilacap	14.39	11.10
Sukabumi City	8.79	11.95
Kebumen	20.44	12.74
Magetan	11.35	12.24
Kendal	11.62	10.80
Garut	12.81	10.99
Pekalongan City	8.09	10.97
Gunung Kidul	21.73	11.74
Temanggung	11.76	11.38
Purwakarta	9.14	11.79
Wonogiri	12.98	13.92
Bojonegoro	15.71	11.70
Blitar	9.97	12.94
Cirebon	14.77	11.92
Grobogan	13.68	12.40
Kulon Progo	21.4	11.15
Nganjuk	12.69	12.38
Demak	14.44	12.16
Pasuruan	10.72	12.29
Banyuwangi	9.17	10.03
Lamongan	15.38	12.28
Tulungagung	8.57	11.06
Madiun	12.54	12.94
Mojokerto City	6.16	9.12
Cilegon City	4.1	10.62
Ponorogo	11.91	12.06
Magelang City	9.05	10.16
Tasikmalaya City	16.28	10.30
Kediri City	8.51	10.65
Salatiga City	5.8	10.78
Kediri	12.91	12.05
Cirebon City	10.36	10.79
Madiun City	4.89	11.34
Jember	11.22	11.46
Sragen	14.86	11.64
Serang City	6.28	9.45
Bandung Barat	12.67	8.59
Karawang	10.37	10.81
Jepara	8.5	10.85
Boyolali	12.45	11.63
Magelang	13.07	9.29
Sukoharjo	9.26	10.41
Semarang	8.15	10.35
Malang	11.53	9.93
Pati	11.95	11.73
Gresik	13.63	10.00
Banyumas	17.52	8.78
Karanganyar	12.46	10.31
Cimahi City	5.84	7.73
Kudus	7.73	10.08
Klaten	14.89	11.28
Jombang	10.79	11.57
Mojokerto	10.57	8.94
Bogor	8.96	8.24
Bandung	8	8.49
Tangerang	5.71	8.86
Bogor City	7.6	7.86
Bantul	16.33	8.67
Bekasi	5.27	8.17
Malang City	4.6	7.10
Depok City	2.4	6.57
Yogyakarta City	8.75	7.82
Surakarta City	10.89	8.31
Sleman	9.46	7.15
Tangerang City	5.04	6.28
Tangerang Selatan City	1.69	6.30
Sidoarjo	6.44	6.78
Jakarta Pusat	4.16	6.31
Semarang City	4.97	6.98
Jakarta Barat	3.64	5.83
Bekasi City	5.46	5.44
Jakarta Utara	5.91	6.80
Jakarta Selatan	3.41	6.80
Bandung City	4.61	5.42
Jakarta Timur	3.24	5.82
Surabaya City	5.82	5.97

## Conclusion

E-commerce data has the potential to predict poverty. Hence, we try to use the machine learning algorithm to model the e-commerce data. Three feature selection algorithms were used to select the best features. Then, support vector regression is used to predict the poverty rate. The experimental results show that using all features cannot guarantee good performance. F-score shows the best result among the three other statistical-based feature selection algorithms evaluated by using RMSE and R^2^. It produces the highest R^2^ value and the lowest RMSE value. This result indicates that a feature selection algorithm can give performance improvement of a machine learning algorithm for poverty prediction. Besides, we found that the CFS feature selection shows an unstable feature rank. Moreover, the weakness of the proposed method still has difficulty in predicting regions with a higher poverty rate. The model shows its advantages which are error minimization compared to other algorithms. Therefore, the performance gap between the SVR model and the other machine learning e.g. K-NN and LR models is quite large. Overall, results show the potential of implementation of e-commerce data, feature selection algorithm, and machine learning algorithm for poverty estimation. Governments, policymakers, and researchers can consider e-commerce datasets as a proxy for socio-economic conditions. The study has limitations because it uses only 1 year of data. Hence, more data are needed to improve the machine learning model's performance. The additional data might produce a better model, especially for underestimated results. For future research, larger data must be utilized for a more accurate poverty model. However, the major limitation of e-commerce data is data accessibility and confidentiality, making it difficult to get.

## Acronyms

Acronyms used in this paper can be seen in Table 8.

**Table 8:** Acronym list.

SUSENAS	A household-based survey was conducted by the Indonesian Central Bureau of Statistics that collects information on socio-economic characteristics such as education, health, family planning, travel information, crime, housing, social protection, and household consumption and expenditure

## Data Availability

Data are not publicly available and can be obtained by contacting the corresponding author if necessary.
